# Supplementary material for: Loganin Attenuates the Severity of Acute Kidney Injury Induced by Cisplatin through the Inhibition of ERK Activation in Mice
Source: Int J Mol Sci. 2021 Jan 31;22(3):1421. doi: 10.3390/ijms22031421 (PMC7866969; doi:10.3390/ijms22031421)
Supplement: Supplementary file 1 [file ijms-22-01421-s001.pdf]

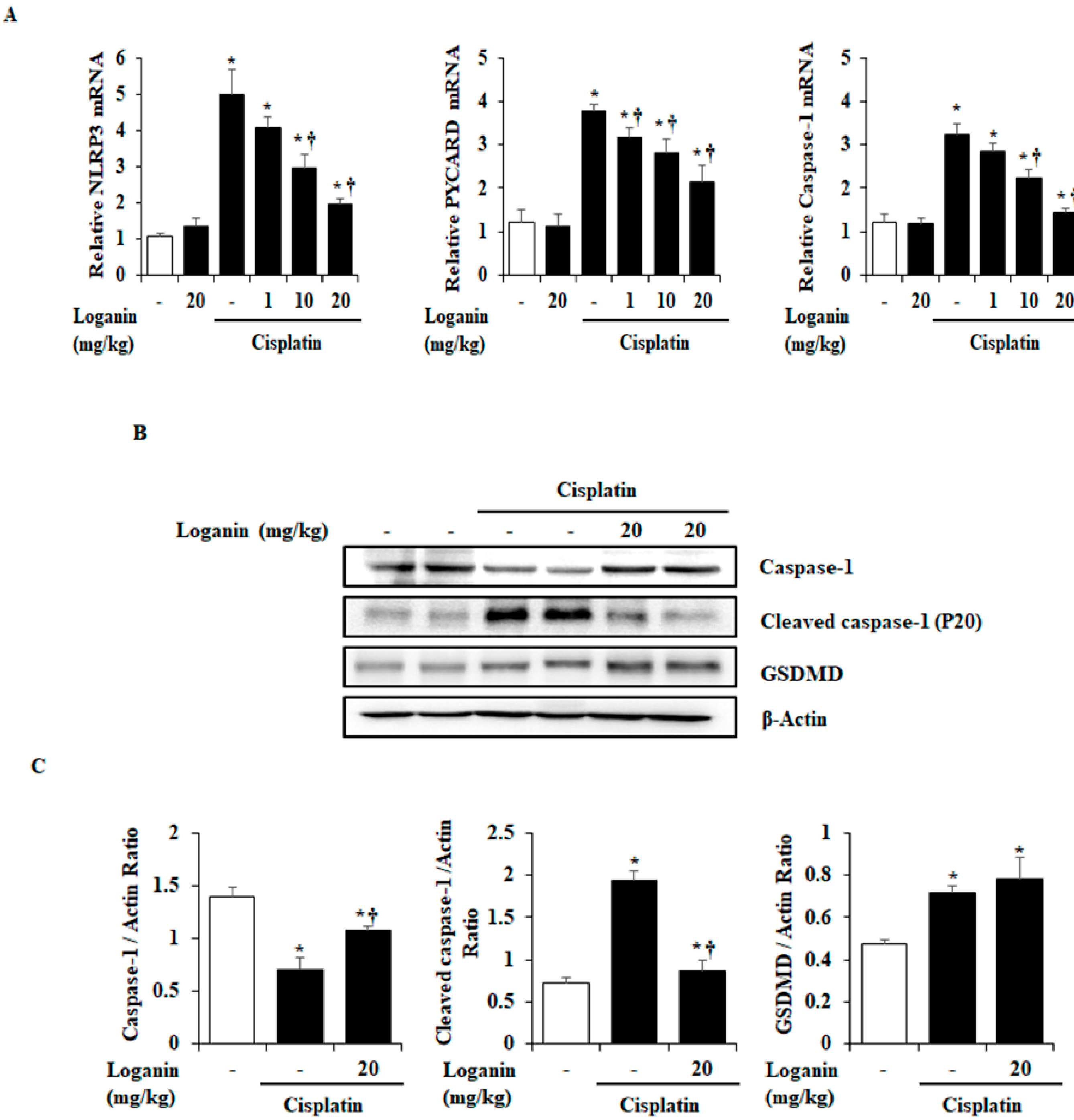

Figure S1. Effect of loganin on renal tubular pyroptosis in cisplatin-induced AKI. (**A**) mRNA levels of NLRP3, PYCARD and caspase-1 were detected by real-time PCR. (**B**) Caspase-1, cleaved caspase-1 (P20) and GSDMD were analyzed by western blot. Actin was used as a loading control. (**C**) The relative density ratio of Caspase-1/Actin, Cleaved caspase-1/Actin, and GSDMD/Actin. Data are represented as mean ± S.E.M. ($n = 9$). (* indicates $p < 0.05$ vs. saline-treated control group, † indicates $p < 0.05$ vs. cisplatin treatment alone).

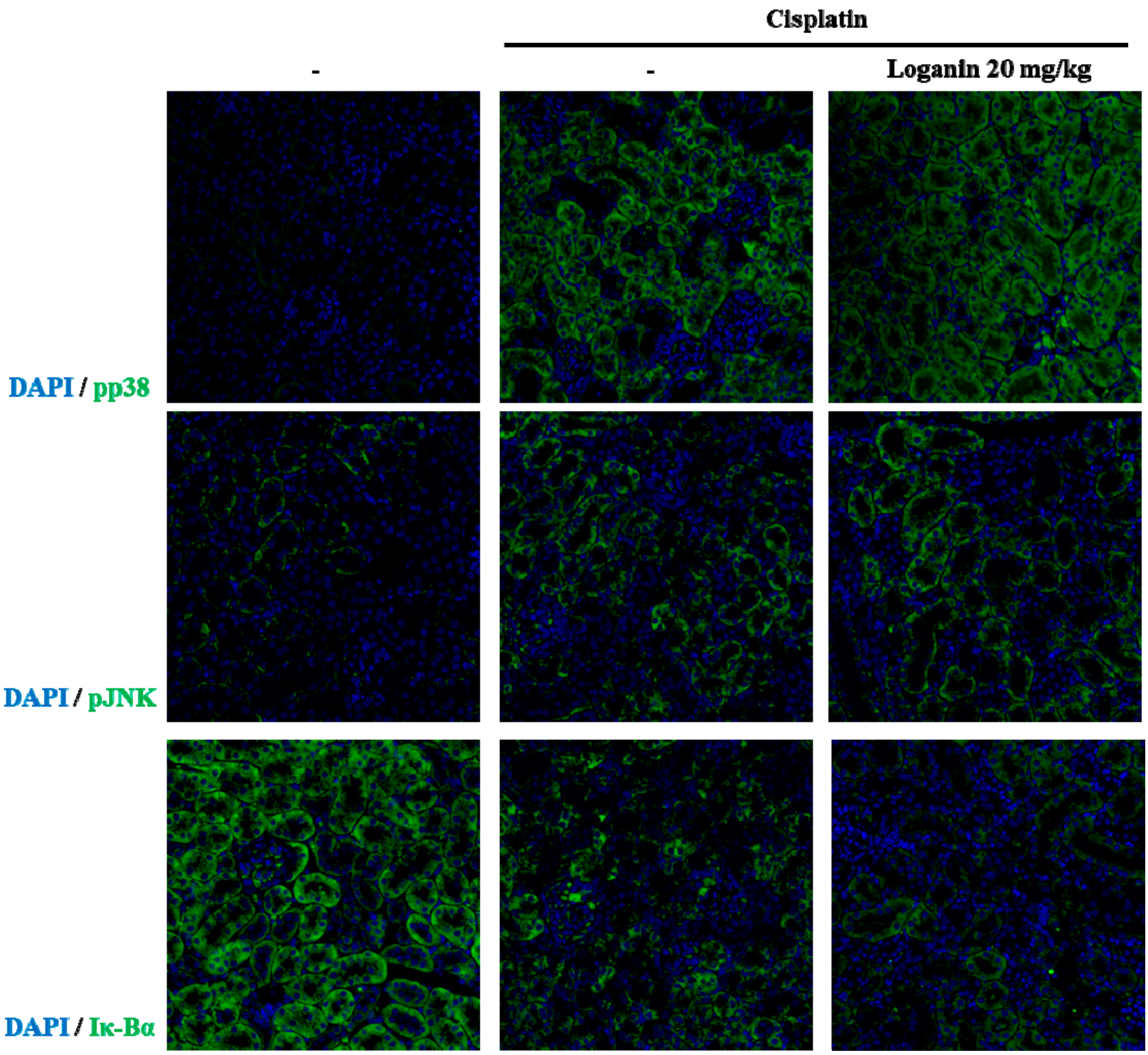

Figure S2. Effect of loganin on phosphorylation of p38, JNK and degradation of Iκ-Bα in cisplatin-induced AKI. Representative pp38, pJNK, Iκ-Bα-stained sections of the kidney (100 × magnification). Each experiment was repeated 3 times.

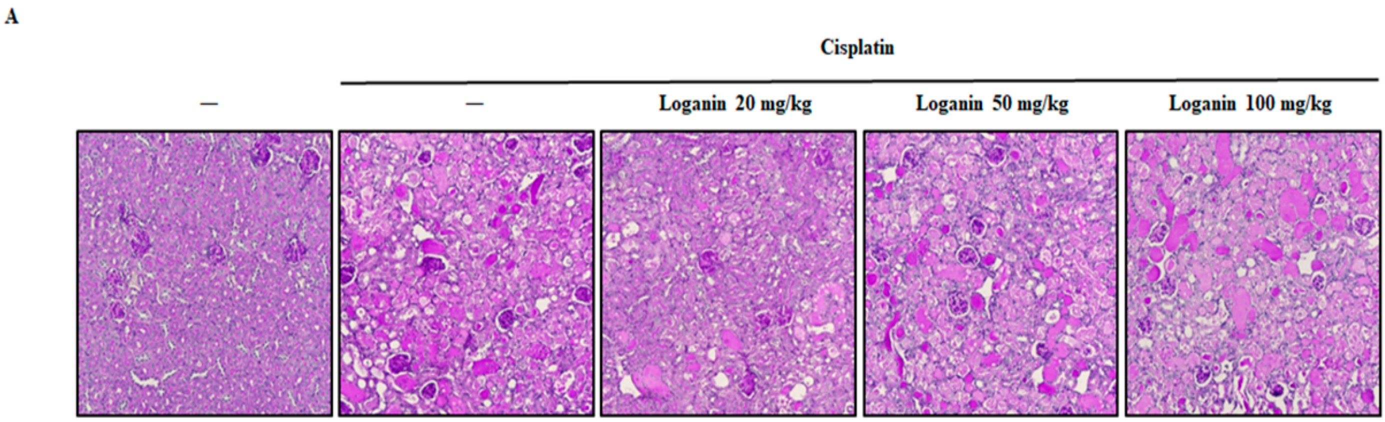

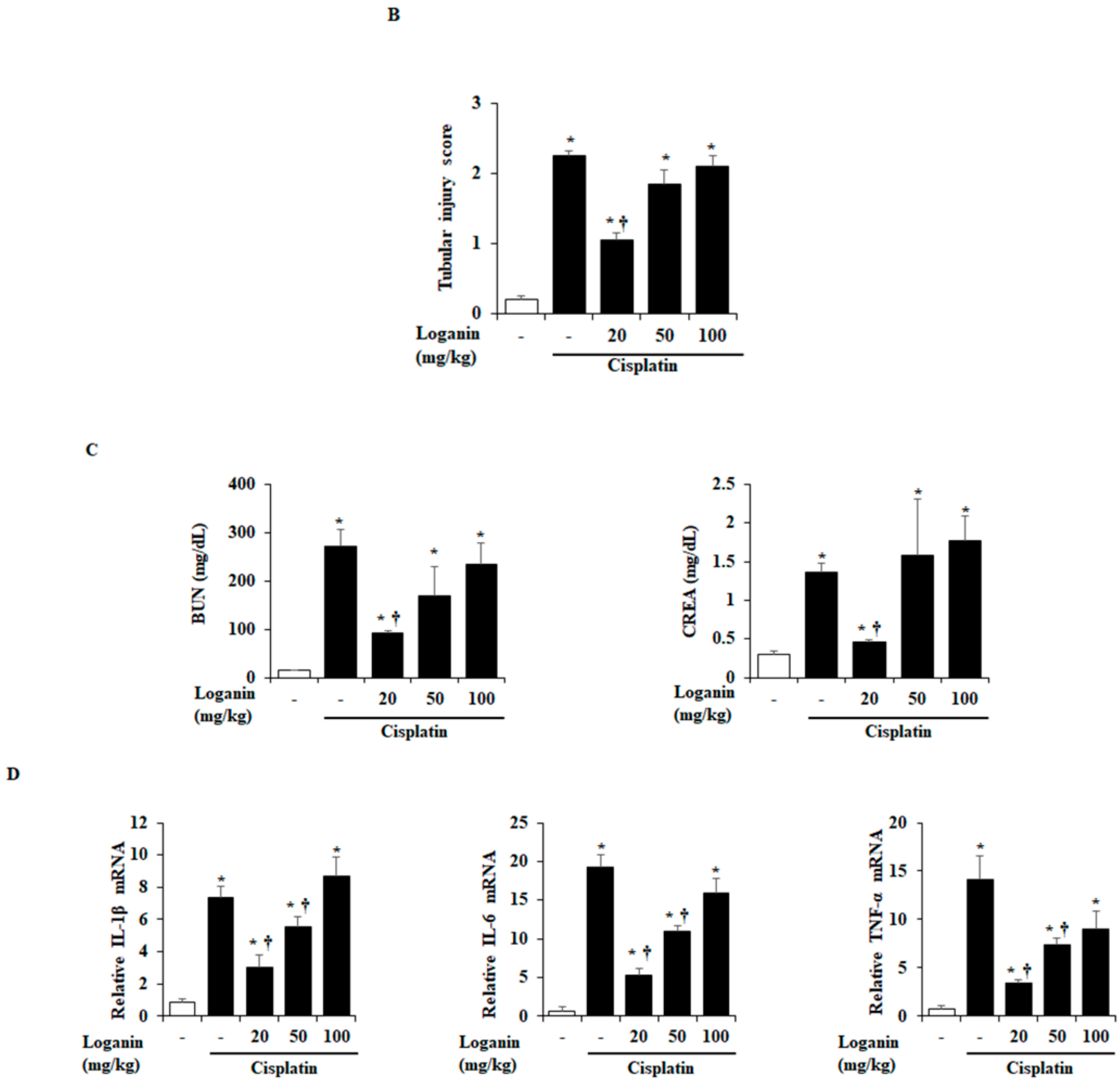

Figure S3. Effects of loganin on the severity of cisplatin-induced AKI. Mice pretreated with loganin (20, 50, or 100 mg/kg) were administered with intraperitoneal injections of cisplatin (10 mg/kg). They were killed at 72 h after the cisplatin injection. (**A**) Representative PAS-stained sections of the kidney (200 × magnification). (**B**) Histological sections of the kidney were scored from 0 (normal) to 4 (severe) for necrosis of tubule cells. (**C**) Serum BUN and CREA were measured at 72 h after cisplatin injection. (**D**) mRNA levels of IL-1β, IL-6, and TNF-α were detected by real-time PCR. Data are represented as mean ± S.E.M. (n = 9). (* indicates $p < 0.05$ vs. saline-treated control group, † indicates $p < 0.05$ vs. cisplatin treatment alone).
